# *BmPMFBP1* regulates the development of eupyrene sperm in the silkworm, *Bombyx mori*

**DOI:** 10.1371/journal.pgen.1010131

**Published:** 2022-03-21

**Authors:** Dehong Yang, Jun Xu, Kai Chen, Yujia Liu, Xu Yang, Linmeng Tang, Xingyu Luo, Zulian Liu, Muwang Li, James R. Walters, Yongping Huang

**Affiliations:** 1 Key Laboratory of Insect Developmental and Evolutionary Biology, Center for Excellence in Molecular Plant Sciences, Shanghai Institute of Plant Physiology and Ecology, Chinese Academy of Sciences, Shanghai, China; 2 CAS Center for Excellence in Biotic Interactions, University of Chinese Academy of Sciences, Beijing, China; 3 Jiangsu University of Science and Technology, Zhenjiang, China; 4 Department of Ecology & Evolution, University of Kansas, Lawrence, Kansas, United States of America; University of Kentucky, UNITED STATES

## Abstract

Sperm deliver the male complement of DNA to the ovum, and thus play a key role in sexual reproduction. Accordingly, spermatogenesis has outstanding significance in fields as disparate as infertility treatments and pest-control, making it a broadly interesting and important focus for molecular genetics research in a wide range of species. Here we investigate spermatogenesis in the model lepidopteran insect *Bombyx mori* (silkworm moth), with particular focus on the gene *PMFBP1* (polyamine modulated factor 1 binding protein 1). In humans and mouse, *PMFBP1* is essential for spermatogenesis, and mutations of this gene are associated with acephalic spermatozoa, which cause infertility. We identified a *B*. *mori* gene labeled as “PMFBP1” in GenBank’s RefSeq database and sought to assess its role in spermatogenesis. Like in mammals, the silkworm version of this gene (*BmPMFBP1*) is specifically expressed in testes. We subsequently generated *BmPMFBP1* mutants using a transgenic CRISPR/Cas9 system. Mutant males were sterile while the fertility of mutant females was comparable to wildtype females. In *B*. *mori*, spermatogenesis yields two types of sperm, the nucleated fertile eupyrene sperm, and anucleated unfertile apyrene sperm. Mutant males produced abnormal eupyrene sperm bundles but normal apyrene sperm bundles. For eupyrene sperm, nuclei were mislocated and disordered inside the bundles. We also found the *BmPMFBP1* deficiency blocked the release of eupyrene sperm bundles from testes to ejaculatory seminalis. We found no obvious abnormalities in the production of apyrene sperm in mutant males, and double-matings with apyrene-deficient *sex-lethal* mutants rescued the *ΔBmPMFBP1* infertility phenotype. These results indicate *BmPMFBP1* functions only in eupyrene spermatogenesis, and highlight that distinct genes underlie the development of the two sperm morphs commonly found in Lepidoptera. Bioinformatic analyses suggest *PMFBP1* may have evolved independently in lepidoptera and mammals, and that despite the shared name, are likely not homologous genes.

## Author summary

The presence of nucleated and anucleated dimorphic sperm produced by a single male is a notable characteristic of lepidopteran insects. Previously we identified the gene *BmSxl* and *BmPnldc1* are required for apyrene and eupyrene sperm development, respectively. However, there remains very little known about the molecular mechanism of eupyrene and apyrene sperm development and function. In human and mouse, the protein PMFBP1 is related to acephalic spermatozoa syndrome. Here, we generate somatic mutants in the silkworm *Bombyx mori* for this gene. In the silkworm, *BmPMFBP1* is essential for male fertility. Loss of *BmPMFBP1* function results in defective eupyrene sperm bundles in the early elongation stage of spermatogenesis. The nuclei of the eupyrene sperm bundles were displaced from sperm heads while the apyrene sperm bundles were normal. This deficiency also results in the failure of the release of eupyrene sperm bundles from testes to the ejaculatory seminalis duct. Our study proves the important function of BmPMFBP1 in the development of the eupyrene sperm in the silkworm and identifies a potential target gene for lepidopteran pest control.

## Introduction

Spermatogenesis is both an interesting and important focus for molecular genetics research. Sperm play a key role in reproduction, and thus aberrant sperm development is a fundamental cause of infertility [1]. Reciprocally, efficiently disrupting spermatogenesis presents notable opportunities for advancing applications as diverse as contraception methods and control of pest species [2,3]. From an evolutionary perspective, the progression of cellular phenotypes through spermatogenesis is generally conserved across animals; even many of the same spermatogenic genes are conserved between insects and mammals [4,5]. Nonetheless, the outcome of this process, spermatozoa, are one the most morphologically diverse cell types known [6]. This makes spermatogenesis a valuable model for understanding how genetics and development interact to produce phenotypes. For all these reasons, there is great value in illuminating the molecular genetic underpinnings of spermatogenesis in a diversity of animal taxa.

One emerging and notable taxon for spermatogenesis research is lepidopteran insects (moths and butterflies) [7–9]. Understanding reproductive processes in this group is important because many lepidopteran species have outstanding economic significance, primarily as agricultural pests, but also due to silk textiles produced from the cultivation of the silkworm moth, *Bombyx mori* [10,11]. Given its exceptional economic status and tractable rearing, *B*. *mori* is the vanguard species for molecular genetic research in Lepidoptera, including for spermatogenesis [12]. Like most other Lepidoptera, *B*. *mori* exhibits the striking phenomenon of dichotomous spermatogenesis, wherein males produce two distinct morphs of spermatozoa [13]. The eupyrene sperm morphs contain a nucleus and DNA while the apyrene morphs lack both a nucleus and nuclear DNA. Both sperm morphs are transferred to females in large numbers during mating, after which only eupyrene sperm fertilize the egg while apyrene sperm assist fertilization by transporting eupyrene sperm to the female sperm storage organs [8,9]. Based on available samples, it appears this dimorphism arose very early in the evolution of Lepidoptera and is currently considered to be a universal feature of all but the most early-diverging lepidopteran lineages, such as the primitive Micropterigidae [14–18].

The cytology and developmental timing of apyrene versus eupyrene spermatogenesis is well-characterized due to detailed microscopic observations, but there currently exists only a rudimentary and piecemeal understanding of the molecular genetic control of this process [9]. Friedlander et al. (2005) [13] provide an extensive review of the developmental cytology, key details from which we summarize here. In testes, spermatogenic cells are partitioned into cysts, which each produce bundles of the same sperm morph. Cysts destined to produce eupyrene versus apyrene sperm can be first distinguished visually at the spermatocyte stage. Eupyrene cells follow a standard spermatogenic trajectory, yielding bundles of sperm with an elongated head containing needle-shaped nuclei and a long tail [19–21]. By contrast, apyrene cells exhibit aberrant chromosomal pairing during meiosis I, followed by the degradation of nuclear DNA, which forms rounded micronuclei towards the center of the elongating spermatid bundles, and is ultimately squeezed out along with excess cytoplasm during spermiogenesis [19,21]. Developmental timing also distinguishes eupyrene versus apyrene spermatogenesis. Eupyrene spermatogenesis begins during early larval stages and ends around the onset of pupation, while apyrene spermatogenesis typically begins just prior to pupation [13].

At present, only a few genes have been experimentally linked to spermatogenesis in *B*. *mori*, primarily through CRISPR gene editing knock-out mutations. Most prominently, mutations in *Sex-lethal* (*BmSxl)* prevent the production of apyrene sperm, but do not alter eupyrene sperm production (nor impact sex determination, as might be expected from this gene’s role in other insects) [8,9]. The reverse pattern was found in silkworms deficient for *BmPnldc1* (*poly(A)-specific ribonuclease-like domain-containing 1*), which caused eupyrene sperm abnormalities [9]. And a third gene, *BmMael* (*Maelstrom*), was essential for spermatogenesis of both morphs, with mutants displaying defective eupyrene and apyrene sperm [22]. Beyond these three genes, little is known concerning the molecular mechanisms of lepidopteran sperm development. Nonetheless, these results suggest substantial partitioning of the genetic control of apyrene versus eupyrene spermatogenesis, and motivate the search for additional relevant genes.

Given previous success in using CRISPR genome engineering to assess a specific gene’s impact on sperm development in *B*. *mori*, and the precedent of conserved spermatogenic genes between insects and mammals, we conducted a literature search to find further candidate genes to investigate [4,22]. One gene we identified was *PMFBP1* (polyamine modulated factor 1 binding protein 1), mutations in which appear to cause acephalic spermatozoa syndrome. Acephalic spermatozoa syndrome, characterized by a teratozoospermia yielding headless spermatozoa in the ejaculate, is heritable and causes severe male infertility in both human and mouse [23,24]. The gene *SUN5* (Sad1 and UNC84 domain containing 5) was initially identified as being responsible for this autosomal-recessive acephalic spermatozoa syndrome [25,26]. But more recently, Zhu et al (2018) discovered that men with homozygous mutations in PMFBP1 also caused a comparable version of acephalic spermatozoa syndrome. These two proteins are localized at the sperm head-coupling and directly interact to maintain the connection integrity of sperm head and tail [27,28].

Through database searches, we identified a gene provisionally annotated as PMFBP1 in *B*. *mori* (hereafter, *BmPMFBP1*), and have subsequently investigated its function in silkworm spermatogenesis, which we report here. We found *BmPMFBP1* was specifically expressed in testes, and we generated *BmPMFBP1* mutants using a binary CRISPR/Cas9 system. Deficiency in *BmPMFBP1* caused male sterility, but the mutation appeared only to impact the development of eupyrene sperm, conspicuously disrupting the localization of nuclei during early elongation stage. Additionally, in mutant males, the eupyrene sperm bundles failed to migrate from testes into the ejaculatory seminalis. Comparative genomic analysis readily identified close homologs of BmPMFBP1 in other Lepidoptera, but not in other insects. Alignments to mammalian PMFBP1 revealed the shared presence of a widely-conserved *structural maintenance of chromosomes* (SMC) domain, but otherwise no detectable similarity between sequences. The differences in mutant phenotypes observed between taxa, together with the general lack of sequence homology, suggest that the lepidopteran and mammalian versions of this gene may have evolved independently. Nonetheless, our functional analysis clearly shows that BmPMFBP1, like its mammalian namesake, is a critical spermatogenesis gene, though with a function in silkworm apparently limited to eupyrene sperm development.

## Results

### Initial characterization of BmPMFBP1

Several recent publications highlighted the critical role of PMFBP1 in mammalian spermatogenesis, suggesting that a homologous sequence in *B*. *mori* would provide a good target for study [27–30]. We subsequently identified a gene in the *B*. *mori* NCBI RefSeq gene set (Version 103) annotated as “polyamine-modulated factor 1-binding protein 1” (NCBI Gene ID: 101744903), the locus we henceforth refer to as *BmPMFBP1*. The RefSeq annotation indicated two isoforms somewhat shorter (722 and 855 amino acids) than the mammalian PMFBP1 sequences (~1000 residues). Both BmPMFBP1 and the mammalian sequences (e.g. Human, NCBI Gene ID: 83449) contain the same centrally located ~300 amino acid SMC structural domain (Conserved Domain Database cluster: cl34174). We also examined gene model predictions in SilkDB3 database, which showed discrepancies from the NCBI RefSeq models [31]. There were four distinctly shorter SilkDB3 gene models in the genomic region corresponding to the full RefSeq model. Given the striking discrepancies in gene models from different annotations, we proceeded to clone the cDNA from RNA in order to directly assess the gene structure. Our resulting full-length cDNA is quite similar to the shorter RefSeq (X2) isoform, but lacks a couple exons (S1 Fig).

**Fig 1:**
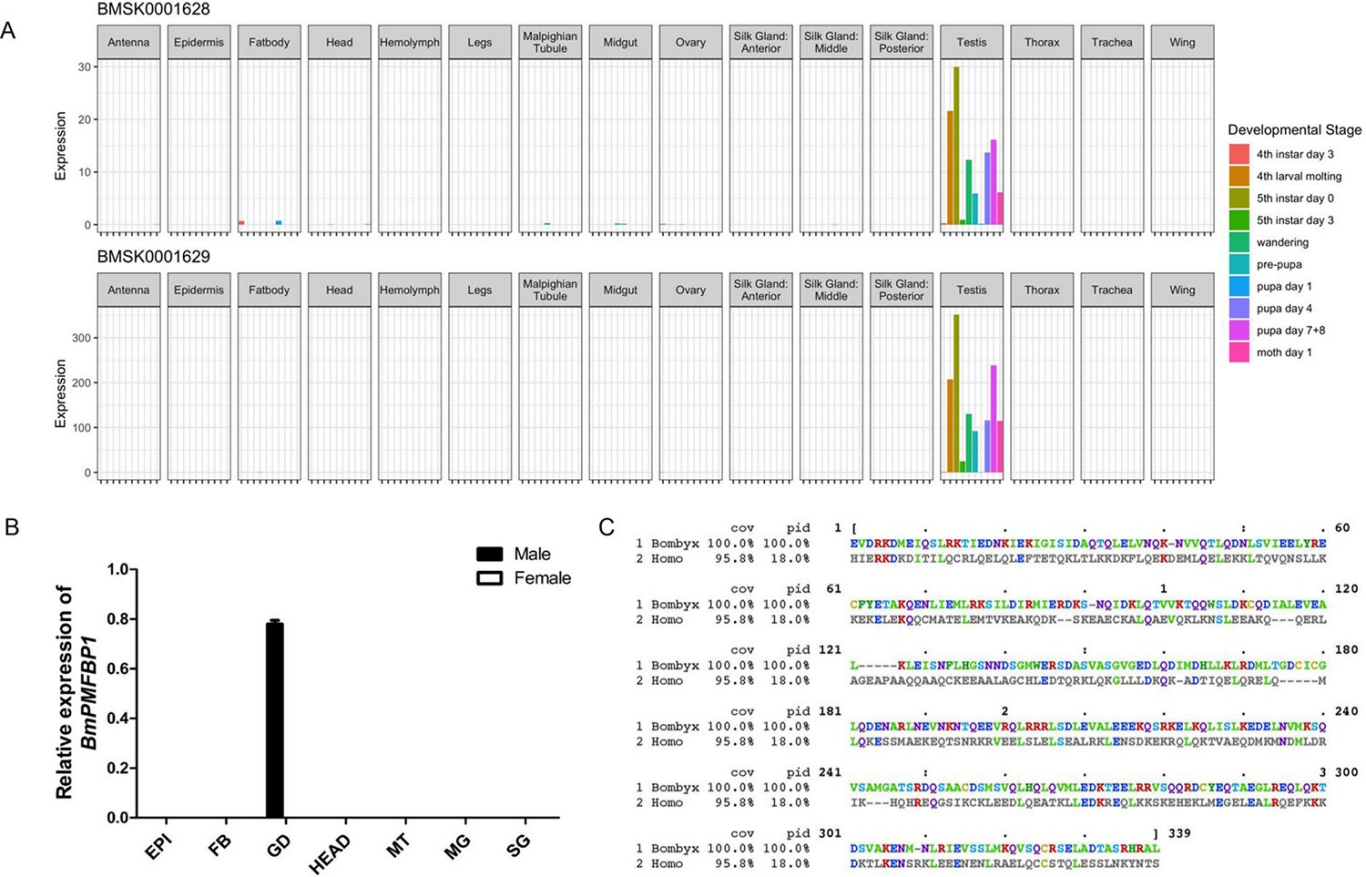
BmPMFBP1 is specifically expressed in testes. (A) Tissue and developmental stage-specific levels of gene expression for two SilkDB3 annotations corresponding to the BmPMFBP1 locus. (B) The relative transcript level of *BmPMFBP1* in various tissues from the fourth day of the fifth larval instar (L5D4). Tissues including epidermis (EP), fat body (FB), gonad (GD, testes in male and ovary in female), head (HE), midgut (MG), Malpighian tubule (MT), silk gland (SG). (C) HMM (hidden Markov model) profile alignment of the SMC domain in PMFBP1 from human (*Homo*) and silkworm (*Bombyx*). Protein regions outside of those represented here failed to align.

Both mouse and human PMFBP1 are primarily expressed in testes, consistent with the protein’s crucial role in spermatogenesis [27,28]. If BmPMFBP1 has a similar role in silkworm, we expect to see a testes-biased pattern of gene expression. We therefore assessed tissue-specific patterns of expression for *BmPMFBP1* using qRT-PCR, as well as the silkworm expression atlas available from SilkDB3 [31]. With qRT-PCR, we assayed expression in the testes, ovaries, and six other somatic tissues of both male and female silkworm. Tissues were sampled on day four of fifth instar larvae, a developmental timepoint occurring within the transition from eupyrene to apyrene spermatogenesis (Fig 1B) [13]. We detected *BmPMFBP1* expression only in testes.

SilkDB3 provides transcript abundances for >80 tissues and developmental stages, but in the case of *BmPMFBP1*, the utility of these data were compromised by the apparently flawed and piecemeal gene models used for estimating expression levels (S1 Fig). Nonetheless, portions of the four SilkDB3 shorter gene models were concordant, at least in part, with portions of the cloned sequence and two appeared to have informative levels of expression reported. Based on patterns of expression from these two gene models, BmPMFBP1 appeared to be expressed robustly and almost exclusively in testes from larvae, pupa, and adults (Fig 1A). Thus, the available expression data from SilkDB3 reinforce our qRT-PCR results in showing a testes-specific expression pattern for *BmPMFBP1*, which together provided further support for the hypothesis that this gene may have a spermatogenic function in male silkworm similar to that in human and mouse.

### PMFBP1 homology

Despite the RefSeq annotation for this *B*. *mori* protein, which is nominally based on homology (https://www.ncbi.nlm.nih.gov/genome/annotation_euk/process/), the extent of sequence conservation or evidence for orthology was unclear between mammals and silkworm (or other insects). Sequences from both taxa share an SMC protein domain, described as a “chromosome segregation ATPase”. However, this “superfamily” domain occurs broadly across eukaryotes and is found in proteins with diverse functions [32]. So, sharing this SMC domain is, at best, a superficial indicator of direct conservation of this particular gene between moths and mammals. Pairwise alignment between Human and silkworm PMFBP1, either via BLASTP or global alignment, fails to identify any regions of similarity other than in the SMC domain (Fig 1C). BLASTP searches of BmPMFBP1 against the Human proteome, and vice versa, return no significant hits (Evalue < .001). The NCBI HomoloGene database lists PMFBP1 (HomoloGene: 23182) as “Conserved within Tetrapods”, indicating no orthology with insect genes. Respectively, orthologs for BmPMFBP1 are reported for several lepidopteran species but not from other insects in OrthoDB (Group 1430682at2759) [33]. Accordingly, none of these computational analyses or resources provide compelling evidence for direct conservation or orthology between mammalian PMFBP1 and its silkworm counterpart that we report on here.

Nonetheless, these analyses largely rely on a “generalized” application of BLAST and related sequence alignment algorithms which can have limited sensitivity to detect very old or remote homologies. A more sensitive assay may be achieved by building a sequence specific position matrix with which to perform homology detection, such as is implemented via hidden Markov models (HMM) in the HMMer software suite [34,35]. Accordingly, we generated an HMM profile based on an alignment of PMFBP1 from over 150 mammal species (S2 Fig) and used this to search insect genomes. Strikingly, when using this custom HMMprofile to search the *B*. *mori* RefSeq proteome, BmPMFBP1 is the top hit. The only other significant hit (E-value < 1e-5) reported is annotated as “Tropomyosin-2”.

Recovering BmPMFBP1 as the top hit in the *B*. *mori* genome using the HMM profile search raises the question whether homologous sequences may similarly be detected in other insects. Direct BLASTP queries with BmPMFBP1 to the *Drosophila melanogaster* proteome returned no significant hits. A subsequent HMMprofile search in *D*. *melanogaster* yielded significant hits only to various isoforms of Myosin heavy chain (Flybase:CG17927) that do not contain an SMC domain. A broader HMMprofile search to insect proteomes in the EnsemblMetazoa database similarly reported hits to proteins annotated as myosin or tropomyosin [36]. So, while orthologs for BmPMFBP1 are readily detected in other moths and butterflies, there is no evidence for closely related sequences in other insect lineages. Unfortunately, the gene models corresponding to BmPMFBP1 in other Lepidoptera are notably piecemeal (like in SilkDB3) and do not readily support constructing a meaningful HMM profile for lepidopteran PMFBP1 with which to perform HMM profile searches outside of Lepidoptera.

We are thus left with a somewhat ambiguous pattern of homology concerning BmPMFBP1 relative to mammalian PMFBP1. There are clearly homologous regions of the proteins between these taxa, specifically in the broadly conserved SMC domain, but regions outside this domain do not align. Established orthology pipelines give no indication that this gene is conserved between insects and mammals, and yet an HMMprofile search specifically recovers BmPMFBP1 as the top hit in the *B*. *mori* proteome. Finally, searching in other insects, either using the mammalian HMMprofile or BLASTP with BmPMFBP1, yields no obvious homologs outside of Lepidoptera. So, while it remains possible that this is the “same” gene (i.e. orthologous) conserved between tetrapods and Lepidoptera, a more plausible scenario may be that this gene originated independently in lepidopteran insects, and its apparent affiliation with mammalian PMFBP1 reflects convergent evolution.

### Constructing *BmPMFBP1* mutants using a binary CRISPR/Cas9 system

**Fig 2:**
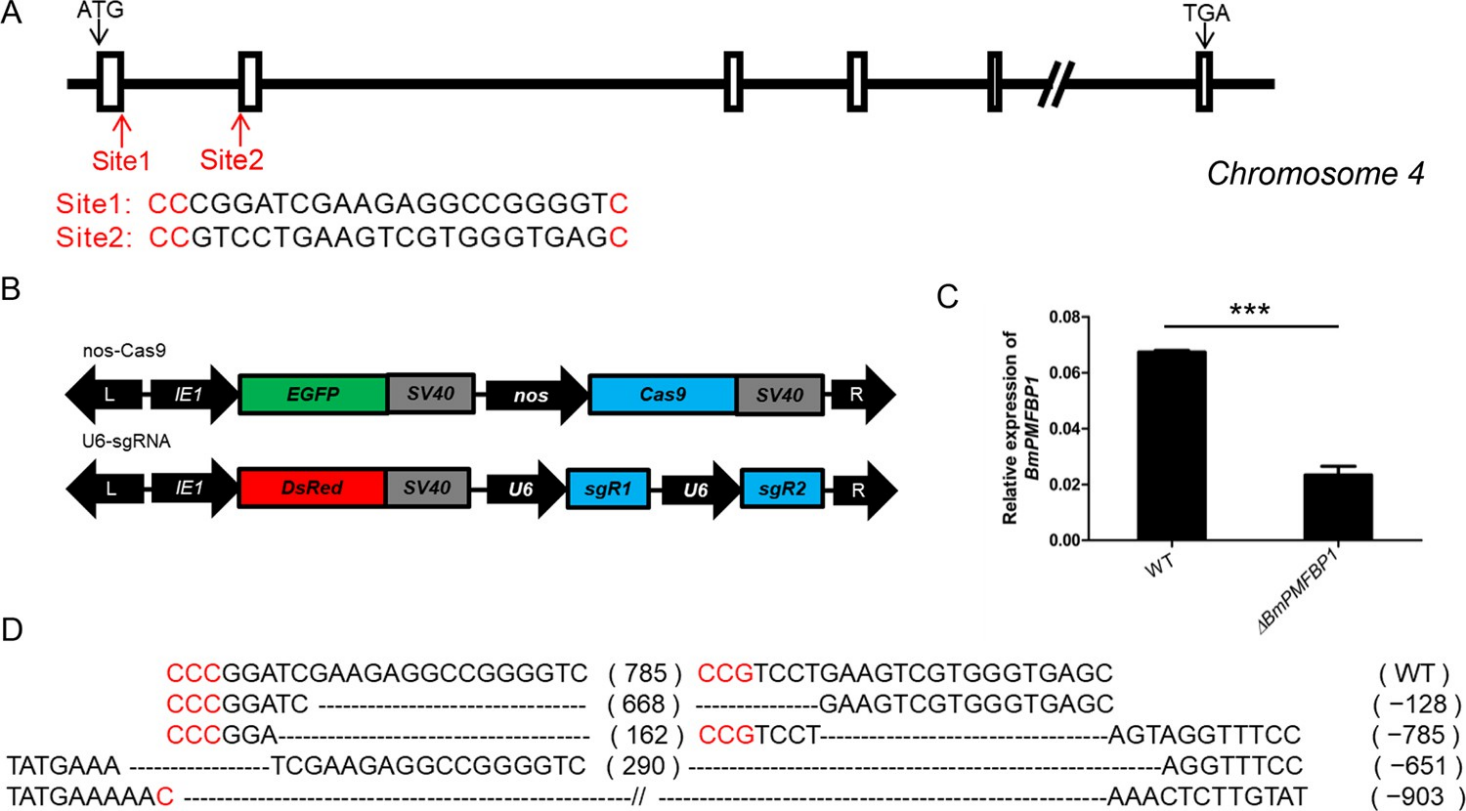
Construction of *BmPMFBP1* mutants using the binary transgenic CRISPR/Cas9 system. (A) Schematic of the *BmPMFBP1* gene structure and sgRNA target sites. The boxes indicate the coding exon, the black arrows indicate the start and stop codon, the red arrows indicate the two target sites located in exons 1 and exons 2. (B) Schematic of the binary transgenic CRISPR system vectors for obtaining *BmPMFBP1* mutants. (C) The mRNA expression level of *BmPMFBP1* in three individual WT and mutant testes at L5D4. The asterisks (***) indicate the significant differences (P < 0.001) relative to WT. (D) Examples of mutations induced by CRISPR/Cas9 system. The sequence of the wildtype is displayed at the top. The dotted lines indicate the deleted residues, the PAM sequence is in red, the number of the nucleotides deleted are shown on the right.

Whether due to conservation or convergence, the similarities detected in sequence and expression between mammalian and silkworm PMFBP1 led us to proceed with functional experiments via CRISPR genome editing. So, we constructed *BmPMFBP1* mutants using a binary CRISPR/Cas9 system. The *BmPMFBP1* gene is located on chromosome 4 and has 17 exons, and we designed two target sites, one each in exon 1 and exon 2 (Fig 2A). The transgenic strain nos-Cas9 uses *nanos* promoter to drive the expression of Cas9, and uses the *IE1* promoter to drive expression of the fluorescent protein gene *EGFP*, which serves as the screening marker. Another transgenic U6-sgRNA strain used two *U6* promoters to drive the expression of two site-specific sgRNAs respectively, and used *IE1* promoter to drive the expression of *DsRed* red fluorescent marker protein (Fig 2B). When these two transgenic lines are crossed, the resulting F1 offspring express both Cas9 and sgRNAs, producing DNA lesions in *BmPMFBP1*, such that independent *de novo* mutation occurs in each individual offspring of the cross. Mutations in randomly selected representative F1 offspring were detected by PCR and sequencing using gene-specific primers, confirming mutations were produced in both male and female individuals (Fig 2D). Such mutations will result in transcripts encoding a non-functional version of the protein due to induced frame-shifts and the likely creation of premature stop codons. Often such mutant transcripts will also be subject to nonsense-mediate decay, which will significantly reduce expression of the target loci [37]. Analysis via qRT-PCR showed the expression of *BmPMFBP1* in the mutants significantly decreased compared with wildtype (Fig 2C). These results indicate that we successfully generated *BmPMFBP1* mutant individuals.

### *BmPMFBP1* is essential for male fertility

**Fig 3:**
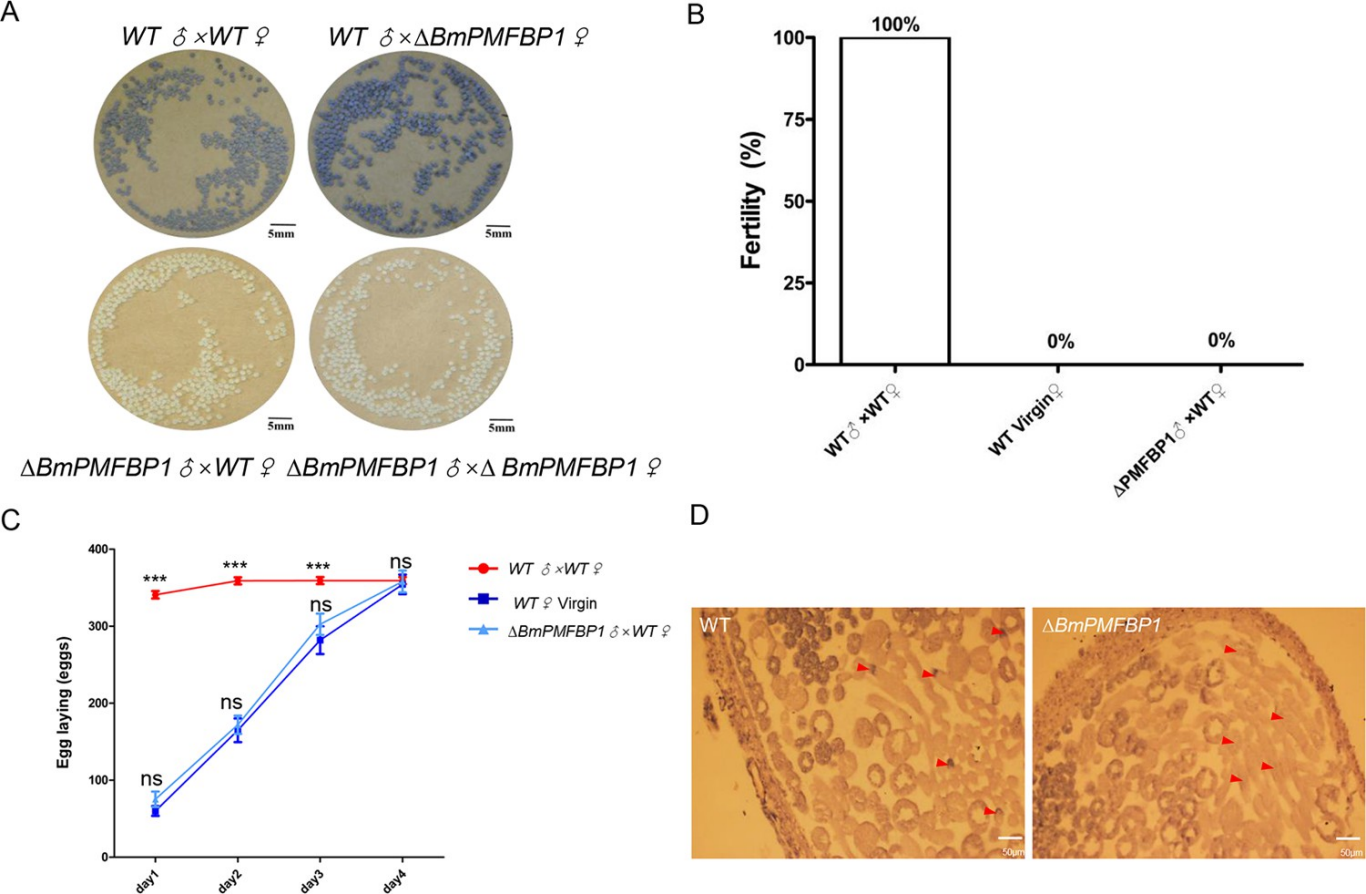
BmPMFBP1 deficiency results male sterility. (A) Photograph of eggs after 8 days laid by wildtype or mutant females mated with wildtype or mutant males. The color of eggs indicated whether the egg is fertilized. Pale yellow eggs are unfertilized, dark black eggs are successfully fertilized. (B) Fertility of *BmPMFBP1* male mutants. Fertility was evaluated as the ratio of fertile individuals to the total number of individuals (n = 20). (C) The cumulative amounts of eggs laid by female. Data are mean ± SEM (n = 30, ns indicates no significant difference compared with WT Virgin, P>0.05; *** indicates significant difference compared with the WT Virgin, P<0.001)). (D) Morphologies of internal structure of wildtype (left) and *BmPMFBP1* mutant (right) testis. The red triangles indicate the nuclei of eupyrene sperm bundles.

In human and mouse, *PMFPB1* is responsible for acephalic spermatozoa syndrome that leads to male infertility [25,26]. To further explore and analyze the biological function of *BmPMFBP1* in the silkworm, we tested the fertility of the mutant. The mutants were viable, and their growth and development did not display obvious abnormality. The fertility tests showed that *ΔBmPMFBP1* caused male sterility (Fig 3A and 3B), while *ΔBmPMFBP1* female fertility was normal (Fig 3A). Daily oviposition patterns of wildtype females mated to *ΔBmPMFBP1* virgin males were indistinguishable from virgin females, but strikingly different from females mated to wild-type males (Fig 3C). Specifically, wildtype virgin females mated with wildtype virgin males laid the vast majority eggs on the first day after copulation (approximately 94% of the total number of laid eggs, 344.44±24.27 eggs, n = 30) with a small number of additional eggs laid on the second and third days. By contrast, wildtype females mated with *ΔBmPMFBP1* virgin males laid eggs at a relatively constant rate over the same four days, a pattern essentially identical to the oviposition behavior of unmated virgin females (Fig 3B and 3C). The eggs of both the unmated wildtype females and the wildtype females mated with *ΔBmPMFBP1* males did not hatch. These results indicate that *BmPMFBP1* is essential for male fertility in *B*. *mori*. Furthermore, the similarity in oviposition and fertility between virgin and wildtype females mated with *ΔBmPMFBP1* males suggests that the *BmPMFBP1* mutation affects the behavior of sperm in the reproductive tract of males, females, or both sexes.

### *BmPMFBP1* deletion causes defects in the development of eupyrene sperm bundles

**Fig 4:**
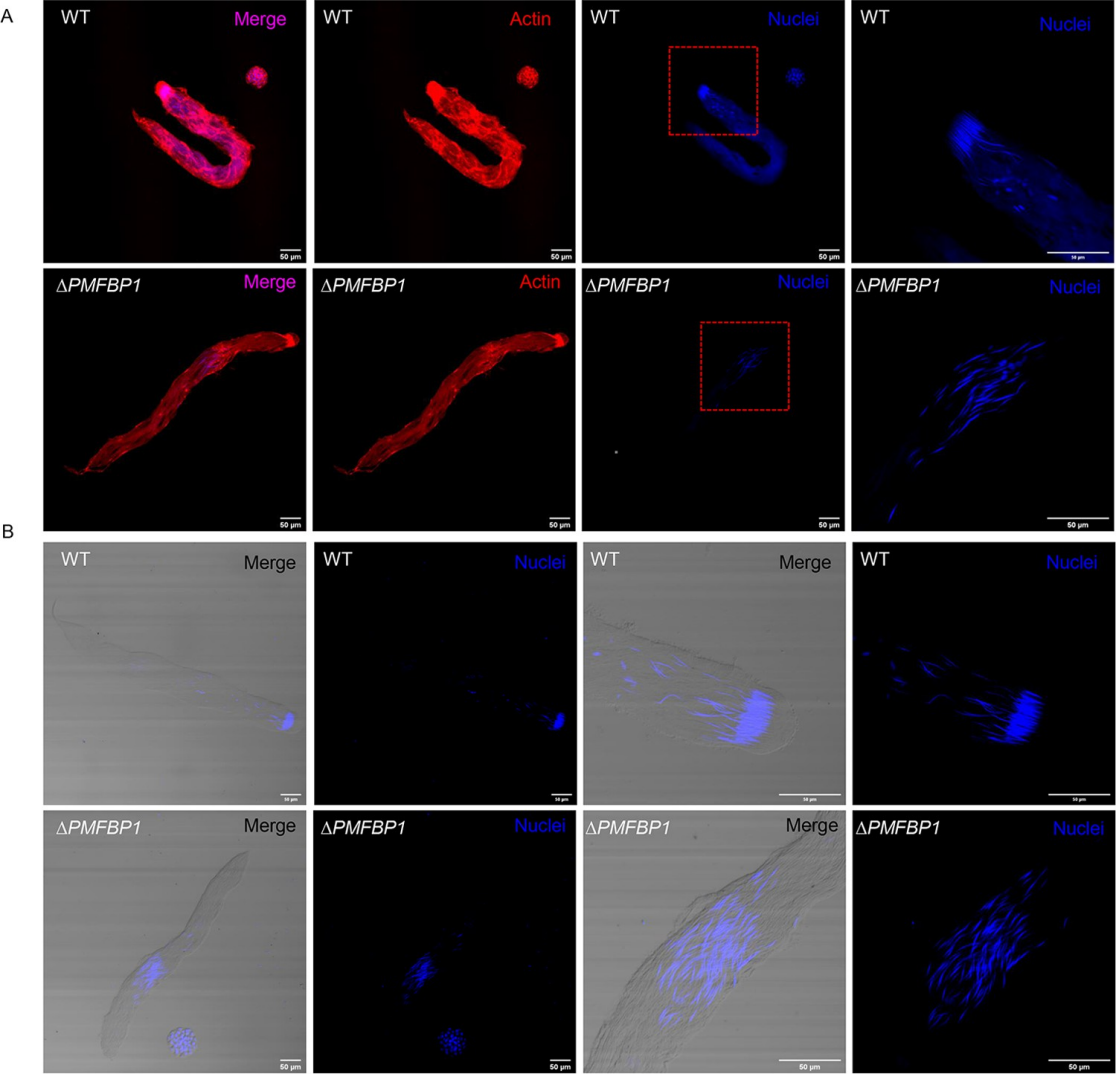
*BmPMFBP1* mutation impairs the development of eupyrene sperm bundles. The eupyrene sperm bundles showed abnormal location and spacing of nuclei in *ΔBmPMFBP1* males. (A) Fluorescence image of eupyrene sperm bundles in testes of wildtype (WT) males and *ΔBmPMFBP1* males on the seventh day of pupal stage. The filamentous actin proteins were stained with TRITC Phalloidin, the nuclei were stained with Hoechst 33258. (B) Additional image of eupyrene bundles with fluorescence staining for nuclei.

We performed further analyses to assess the cause of *ΔBmPMFBP1* male sterility. Since mutations in *PMFBP1* caused acephalic spermatozoa in human and mouse [25,26], we examined the morphology of sperm bundles in the *ΔBmPMFBP1* male silkworms. Firstly, we observed the morphology of testes on the fourth day of the fifth larval stage (L5D4). As reported for mouse, the testis size of mutants versus wildtype displayed no obvious differences (S3A Fig). Histological examination by paraffin section and hematoxylin eosin staining revealed that the structure and morphology of testis were similar with wildtype (S3B Fig). However, mutants showed abnormal positioning of sperm nuclei, which were diffuse and scattered in sperm bundles; in wildtype males the nuclei were tightly concentrated at the heads of the sperm bundles (Fig 3D). We then used fluorescence staining to examine the development of sperm bundles on day seven of the pupal stage. The eupyrene sperm bundles showed significant abnormalities in *ΔBmPMFBP1* males compared to wildtype, but the apyrene sperm did not. For eupyrene sperm, wildtype bundles had an elongated head containing tightly clustered needle-shaped nuclei, but in mutants the nuclei were diffusely scattered in the middle to anterior-half of sperm bundles (Fig 4). Apyrene sperm bundles appeared normal in both wildtype versus *ΔBmPMFBP1* mutant, with round micronuclei that were concentrated in the middle region of the bundles. Examining sperm bundles in testes isolated from adults yielded comparable results, indicating the developmental anomalies in eupyrene sperm caused by *BmPMFBP1* mutations persist throughout development (S5 Fig).

**Fig 5:**
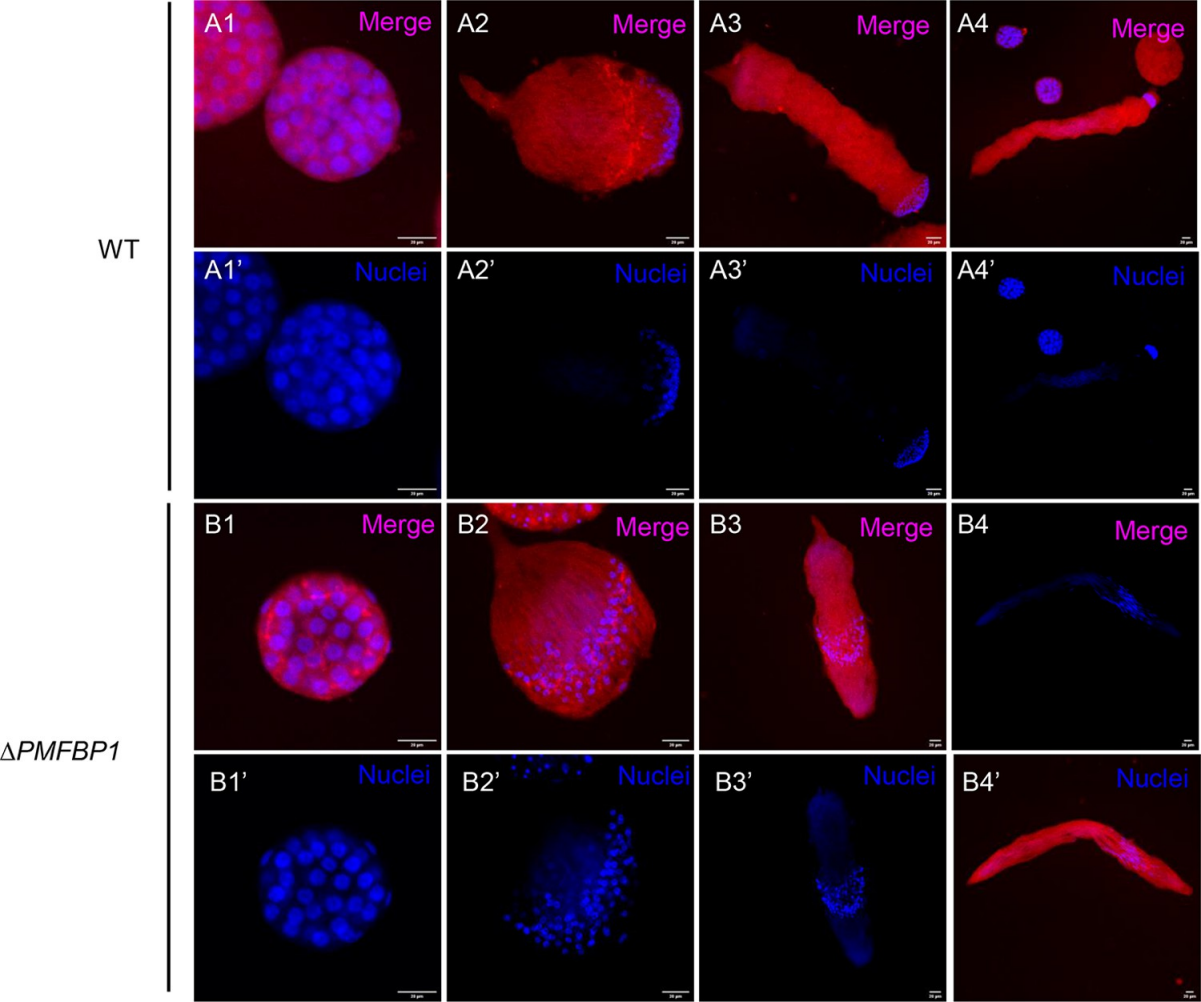
*BmPMFBP1* deficiency causes abnormal development in eupyrene sperm bundles beginning at early elongation stage. (A1-B4’) Fluorescence microscopic images of eupyrene sperm bundles in testes of wildtype (WT) males and *ΔBmPMFBP1* males at L5D4. Images represent the transformation from round spermatid cells (1) through the elongation process to generate bundles of nearly mature spermatozoa (4). The filamentous actin proteins were stained with TRITC Phalloidin, the nuclei were stained with Hoechst 33258.

Eupyrene spermatogenesis occurs before apyrene spermatogenesis. Eupyrene sperm bundles start to appear and elongate during the fifth larval stage, while apyrene sperm bundles initially appear only during the wandering stage [16]. So we investigated spermiogenesis on L5D4, where only eupyrene sperm bundles are produced. In the wildtype, all nuclei gathered in the anterior part and were transformed to a spearhead shape in the elongation stage (Fig 5A). In the mutants, the nuclei similarly underwent the transformation to a spearhead shape, but exhibited diffusely scattered positioning in the middle of eupyrene sperm bundles (Fig 5B). These defects appeared at the beginning of elongation stage (Fig 5A2 and 5B2), and continued through the remaining developmental stages of eupyrene sperm bundles.

In summary, our investigation of sperm developmental processes indicates that the deletion of *BmPMFBP1* causes defects in eupyrene sperm bundles that disrupt the cellular location and structure of nuclei during spermiogenesis, and that these defects start to occur at the early elongation stage. Deletion of *BmPMFBP1* does not have any impacts on apyrene sperm, so far as we could detect.

### *BmPMFBP1* deletion affects the migration of eupyrene sperm

We further investigated the impact of BmPMFBP1 deletion on sperm migration from the testes and transfer to females. To provide context for our observations, we start by summarizing what occurs under typical circumstances [13]. This migration begins with spermiation, the process where spermatozoa are released from the testes into the male genital duct. Spermiation initiates with apyrene sperm bundles being released from the testes prior to eupyrene bundles. Apyrene bundles released from the testes into the vas deferens immediately dissociate into individual apyrene sperm, but eupyrene sperm remain in bundles through spermiation and in the male reproductive tract. Next, the apyrene sperm migrate to the ejaculatory seminalis earlier than eupyrene sperm bundle. During copulation, both types of sperm mix in a single spermatophore that is formed in the female’s bursa copulatrix, at which point eupyrene sperm bundles fully dissociate while the apyrene sperm acquire motility necessary to facilitate eupyrene sperm migration to the spermatheca, from which fertilization can proceed.

**Fig 6:**
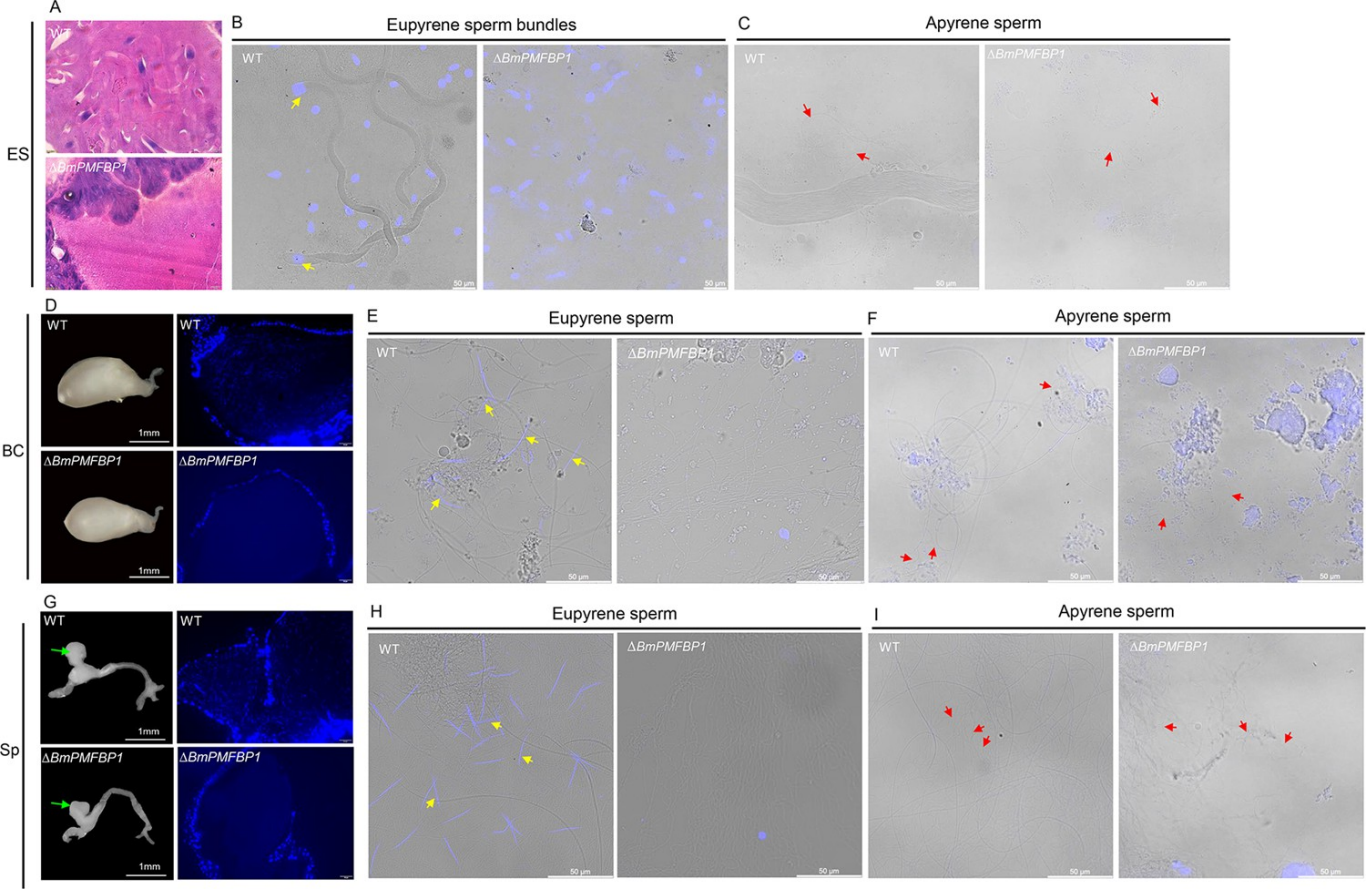
*BmPMFBP1* is essential for eupyrene sperm migration to female reproductive tracts. (A) Morphologies of internal structure of ejaculatory seminalis of unmated WT and *ΔBmPMFBP1* males. (B, C) The smear of the ejaculatory seminalis (ES). (D) Morphologies and internal structure of bursa copulatrix in females mated with WT and *ΔBmPMFBP1* males. (E, F) The smear of the bursa copulatrix (BC) (G) Morphologies and internal structure of spermatheca in females mated with WT and *ΔBmPMFBP1* males. The green arrows indicate the spermatheca. (H, I) The smear of the spermatheca (Sp). The yellow arrows indicated the eupyrene sperm/sperm bundles, the red arrows indicated the apyrene sperm. Paraffin-embedded sections were stained with hematoxylin, eosin, and Hoechst 33258.

To assess how this process of sperm migration and transfer may differ in Δ*BmPMFBP1* males, we observed the behavior of spermatozoa in male and female reproductive tracts. We found the wildtype females mated either with wildtype males or with *BmPMFBP1* male mutants both had a plump bursa copulatrix and spermatheca (Fig 6D and 6G). This indicated that sperm were being transferred to female reproductive tracts, but we couldn’t discern whether apyrene sperm and eupyrene sperm were both being transferred. We used paraffin sections and DAPI staining to examine the internal morphology and contents of these female reproductive organs. We observed nuclei of eupyrene sperm in the bursa copulatrix and spermatheca of wildtype females mated with wildtype males (Fig 6D and 6G). We also observed the smears of the bursa copulatrix and spermatheca of wildtype females mated with wildtype males. We found both eupyrene and apyrene sperm in the bursa copulatrix and spermatheca of wildtype females mated with wildtype males (Fig 6E, 6F, 6H and 6I). In contrast, we did not detect nuclei inside the bursa copulatrix and spermatheca of wildtype females mated with male mutants, indicating the absence of eupyrene sperm inside these tissues (Fig 6E and 6H). And we observed only apyrene sperm on the smears of the bursa copulatrix and spermatheca of wildtype females mated with the mutant males (Fig 6F and 6I). Thus, it appears that only apyrene sperm are present in the plump bursa copulatrix and spermatheca of the females mated with Δ*BmPMFBP1* males. This observation further supports the idea that *BmPMFBP1* deficiency only affects the development of eupyrene sperm, but not apyrene sperm.

We next examined the internal morphology of the ejaculatory seminalis of virgin wildtype and Δ*BmPMFBP1* adult males, in order to discern whether the *BmPMFBP1* mutation impeded the migration of eupyrene sperm from testis into the ejaculatory seminalis or, alternatively, disrupted the migration of eupyrene sperm from the male ejaculatory seminalis to the bursa copulatrix in female. In the testes of adult Δ*BmPMFBP1* males we observed eupyrene sperm bundles (with aberrant nuclei) along with apyrene sperm bundles (S5 Fig). But in the ejaculatory seminalis of Δ*BmPMFBP1* males, we did not find eupyrene sperm bundles; in the wildtype ejaculatory seminalis, we observed numerous eupyrene sperm bundles (Fig 6A–6C). Thus, it appears the *BmPMFBP1* mutation blocked the eupyrene sperm bundles migrating from the testes to the ejaculatory seminalis, which lead to male sterility.

All of our observations via microscopy indicated that deficiency of *BmPMFBP1* had no impact on apyrene sperm development or function. To further assess the functionality of apyrene sperm in *BmPMFBP1* mutants, we performed double copulation experiments using *BmSxl* mutants, which have functional eupyrene sperm but lack functional apyrene sperm [9]. We found that this double copulation rescued the infertility caused by the defective eupyrene sperm in the *BmPMFBP1* mutant males (S4 Fig). This outcome offers further support that apyrene sperm remain unimpacted by mutation of *BmPMFBP1*.

### *BmPMFBP1* is located in the cytoplasm in BmN cells

**Fig 7:**
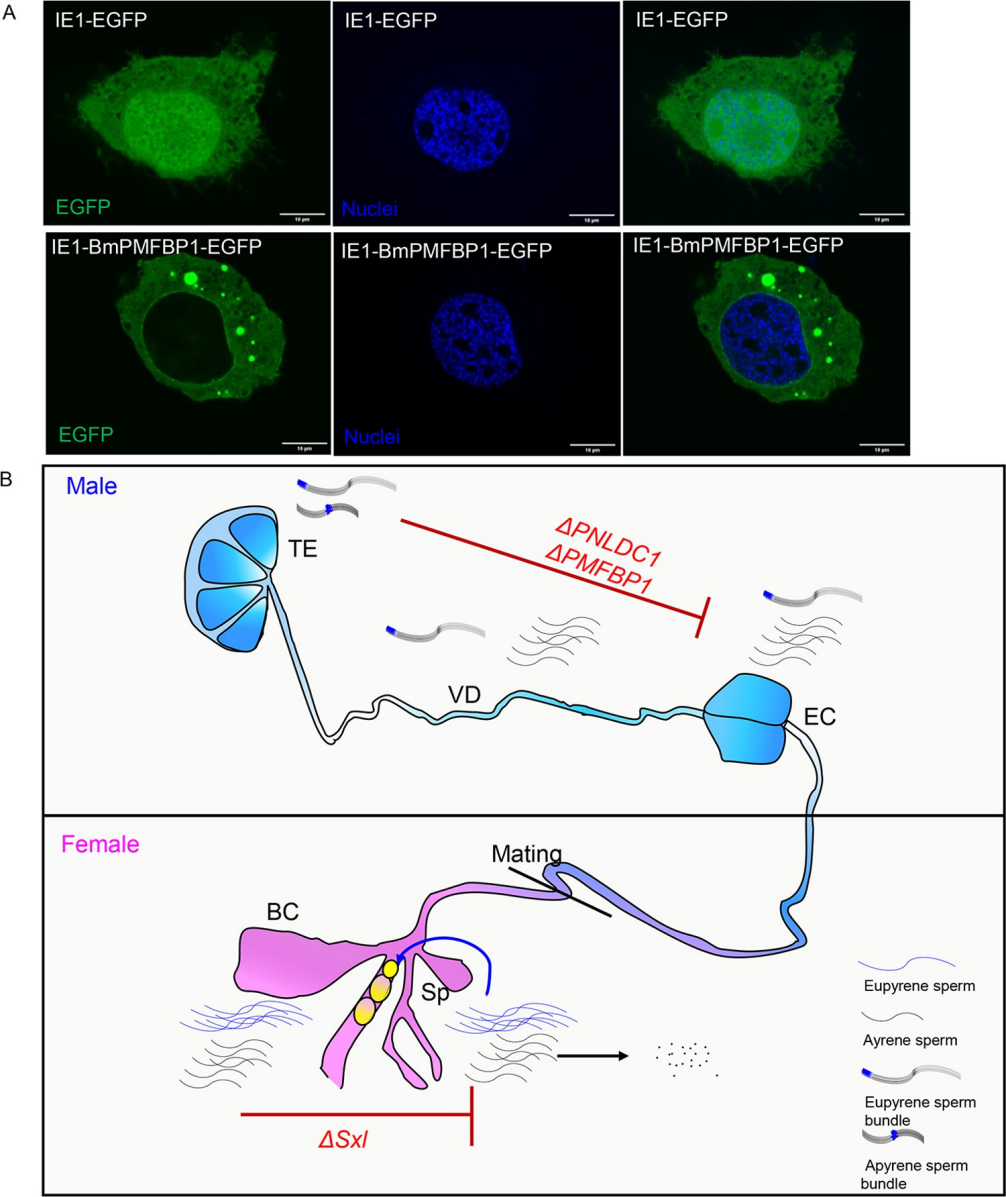
The cellular location of the BmPMFBP1 protein and a schematic of sperm transfer. (A) The cellular location of the fusion protein in BmN cells. (B) Schematic of sperm transferred from the male to female reproductive tracts. The mutation of *BmPNLDC1* or *BmPMFBP1* prevents the migration of eupyrene sperm bundles from the testes to the ejaculatory seminalis. Mutation of *BmSxl* blocks the development of apyrene sperm, which are required to facilitate eupyrene sperm migration from bursa copulatrix to spermatheca. ES: ejaculatory seminalis, VD: vas deferens, BC: bursa copulatrix, Sp: spermatheca.

While SMC domain-containing proteins are generally thought to interact with DNA, and thus be present in the nucleus, this is not the case for mammalian PMFBP1 [27,38]. This raises the question of where BmPMFBP1 occurs in the cell, and whether it localizes to the nucleus or is limited to the cytoplasm. Knowing the cellular distribution of BmPMFBP1 is also a key clue in understanding its role in eupyrene sperm formation and could allow testing the hypothesis convergent evolution in spermatogenesis. An antibody for BmPMFBP1 is not currently available for direct visualization, so we instead used an EGFP fusion protein expressed in a cell line. Specifically, we constructed aplasmid that expressed the BmPMFBP1-EGFP fusion protein driven by an *IE* promoter to assess cellular location. As a control, we used a plasmid driving EGFP alone, without BmPMFBP1. We transfected these plasmids into silkworm BmN cells and observed the distribution of the fusion protein. In the control, EGFP was observed both in the nucleus and cytoplasm (Fig 7A). However, the BmPMFBP1-EGFP fusion protein was observed in cytoplasm only (Fig 7A).

This cellular location of BmPMFBP1 is consistent with observations of PMFBP1’s cytoplasmic location in the round spermatids in the mouse [27]. In mature spermatozoa, mammalian PMFBP1 is a component of the sperm head-to-tail coupling apparatus assembly formed with interacting proteins CENTILEIN and SUN5 [39], and so is specifically localized to the fossa region of the sperm nucleus during sperm head elongation and differentiation [27]. However, if BmPMFBP1 similarly interacts with other sperm proteins, our fusion protein assay would be unlikely to detect it. This is because the BmN cell line is derived from ovaries and presumably does not express other sperm genes that might directly interact with BmPMFBP1. Given that mammalian PMFBP1 and BmPMFBP1 are both limited to the cytoplasm, despite having SMC domains, it seems reasonable to speculate that the silkworm protein similarly functions in some larger structure complex of interacting proteins, like its mammalian counterpart.

## Discussion

In this study, we examined the biological function of the gene *BmPMFBP1* in *B*. *mori*. This locus initially drew our attention while looking for candidate genes related to spermatogenesis in Lepidoptera because its GenBank RefSeq annotation suggested homology to PMFBP1 in mammals. PMFBP1 in mammals was recently shown, through implication in acephalic spermatozoa syndrome, to be a necessary structural protein in sperm [27,28]. While the nature of homology with mammalian PMFBP1 remains ambiguous (see discussion below), our results indicate unequivocally that *BmPMFBP1* does play an essential role in eupyrene spermatogenesis, and is required for male fertility in *B*. *mori*.

As in mammals, we found that *BmPMFBP1* was primarily expressed in testes. We then used a transgenic CRISPR/Cas9 system to disrupt the function of *BmPMFBP1*. While mutant females appeared unaffected, the male mutants were completely sterile, which appeared to result from disruption of eupyrene spermatogenesis. Particularly, our investigations identified two major facets of eupyrene spermatogenesis in which *ΔBmPMFBP1* males differed from their wildtype counterparts. First, we observed during spermiogenesis in mutant males that the nuclei were mislocated in the sperm cells; this was accompanied by a malformed head cyst in the sperm bundle. Second, we observed the absence of eupyrene sperm in the ejaculatory seminalis, which suggests a failure of spermiation such that eupyrene sperm bundles do not effectively migrate from the testes to the downstream regions of the male reproductive tract. Consequently, eupyrene bundles are not passed to females during mating, thus rendering *ΔBmPMFBP1* males sterile. Notably, so far as we have investigated, these mutations causing defects in eupyrene spermatogenesis do not appear to be affecting apyrene sperm. The *ΔBmPMFBP1* males still appear to produce and transfer apyrene sperm to females in a manner comparable to wildtype males, and these apyrene sperm are sufficient to recover fertility in double-matings with *BmSxl* mutants which are sterile due to lacking apyrene sperm. Nonetheless, given that we have not yet exhaustively investigated apyrene spermatogenesis in *ΔBmPMFBP1* males, it is premature to conclude that apyrene spermatogenesis is completely unaffected by mutations in *BmPMFBP1*.

While these results establish that BmPMFBP1 plays a critical role in eupyrene spermatogenesis, what precisely that role is remains unclear. The *BmPMFBP1* mutant phenotypes are not obviously analogous to the phenotypes observed in mammals. For instance, in mice, PMFBP1 is required to physically connect sperm heads and tails. In mutant mice, sperm tails become separated from heads during late-stage spermiogenesis, yet spermiation proceeds, resulting in substantial quantities of acephalic sperm tails observed in the epididymis [27]. In the case of silkworm *BmPMFBP1* mutants, spermiation apparently fails for eupyrene sperm bundles, as they are not observed in the ejaculatory seminalis. Deformation and displacement of the nuclei during spermiogenesis, as we observe in mutant silkworms, is not reported in mammals. And at no point did we observe acephalic spermatozoa in *BmPMFBP1* mutant silkworm. These differences in mutant phenotype do not strongly suggest comparable roles the mammalian and silkworm proteins. However, there is one notable caveat concerning the lack of acephalic sperm in the silkworm mutants. Eupyrene sperm develop in bundles throughout spermiogenesis, which physically constrain them and could possibly prevent separation of heads from tails even if no longer attached. Furthermore, we note that the sperm bundle head-cyst was deformed in *ΔBmPMFBP1* males. Thus, it remains plausible that there is more in common between the mammalian and moth mutant phenotypes than our current results may indicate.

At the level of protein structure, the only detectable similarity between mammalian and silkworm sequences is the SMC domain. However, even the presence of this protein domain is not a clear indication of shared protein function. While SMC proteins primarily function in complexes that serve to model and maintain chromatin structure, there are notable exceptions. For instance, the gene *SMC3* produces a protein forming part of the cohesion complex which binds sister chromatids [38]. Yet the same protein, heavily glycosylated and extracellularly secreted (and referred to as bamacan), is an important component of the extracellular matrix [32,40]. It seems apparent that PMFBP1 in mammals does not follow the canonical role of SMC proteins, given that it specifically occurs outside the nucleus at the head-tail junction in sperm [27]. By contrast, the observation of deformed, dislocated nuclei in *ΔBmPMFBP1* males would initially seem consistent with a protein that functions primarily in controlling chromosomal conformation and condensation. There is a clear precedent for SMC proteins playing an essential role in organizing chromosome structure during spermatogenesis in both mammals and insects [41,42]. However, expressing EGFP-BmPMFBP1 in *Bombyx* cells clearly showed the protein was in the cytoplasm but absent from the nucleus. This observation is inconsistent with a role in controlling chromosomal structure, but does align with potentially having a structural function similar to the mammalian PMFBP1 protein.

Despite sharing an SMC domain, as well as a given name, it remains difficult to confidently characterize the evolutionary relationship between mammalian and lepidopteran versions of PMFBP1. The fundamental question here is whether or not these genes are orthologous (or even paralogous). If they are, then we would conclude that they have been maintained in the genome, and perhaps even with a shared spermatogenic function, since the common ancestor of mammals and moths. The results of our HMM searches, parameterized from mammalian sequences and obtaining BmPMFBP1 as the top hit in silkworm proteome, suggest this is plausible. This would represent a very deep evolutionary conservation, and there is certainly precedent for this among SMC-containing proteins [4,38]. But in such cases, we would also expect to detect orthologs in many taxa that originated from that common ancestor, which would include all insects. Yet our own searches as well as established orthology databases do not reveal any indication of PMFBP1 occurring among insects outside of Lepidoptera, pointing distinctly to the possibility that the lepidopteran version of this protein arose independently, somehow convergently acquiring or “coopting” an SMC domain along the way. If BmPMFBP1 is a novel gene–apparently a synapomorphy among moths and butterflies–then the testes-specific expression and any shared function in spermatogenesis must be convergent with mammals.

Assuming that BmPMFBP1 arose uniquely in Lepidoptera, then its origin could be tied to the origin of dichotomous spermatogenesis and apyrene sperm in Lepidoptera. We currently have very little knowledge concerning the genetic underpinnings of dichotomous spermatogenesis in Lepidoptera (or any other taxa, for that matter). With the addition here of BmPMFBP1, four genes are now known directly to impact spermatogenesis in silkworm. Knocking out *Maelstrom*, a gene in the piRNA-pathway, disrupted both apyrene and eupyrene sperm alike [22]. However, knockouts for the other three loci had phenotypes that appear to be sperm-morph specific. Loss of *sex-lethal* disrupted apyrene sperm development [8,9]. But both BmPMFBP1 and BmPNLDC1, when mutated, appear to impact only eupyrene sperm. Yet the specific phenotypes associated with each are distinct, as are the likely molecular functions of these two genes. BmPNLDC1is a pre-piRNA trimmer, and its loss of function primarily impacted the shape of eupyrene nuclei [9]. By contrast, it is primarily the location of eupyrene nuclei that is disrupted for *ΔBmPMFBP1* males, and these defects appeared earlier in the elongation stage than did the nuclear defects associated with BmPNLDC1. While the molecular function of the SMC-containing BmPMBFP1 is still unknown, it seems unlikely to involve piRNAs, and so taken together, this set of genes clearly points to a diversity of relevant regulatory and molecular mechanisms governing dimorphic spermatogenesis in Lepidoptera.

## Materials and methods

### Silkworm strain

Nistari, a multivoltine, nondiapausing silkworm strain, was used in this study for all experiments. The larvae were feed on fresh mulberry leaves under standard 25°C rearing conditions.

### CRISPR/Cas9-mediated construction of mutants

A binary transgenic CRISPR/Cas9 system was used to construct Δ*BmPMFBP1*. The transgenic strain nos-Cas9 (*IE1*-*EGFP*-*nos*-*Cas9*), expressing Cas9 protein, was constructed previously [43]. The transgenic strain U6-sgRNA (*IE1*-*DsRed*-*U6*-*sgRNA1*-*U6*-*sgRNA2*) uses the U6 promoter to drive the expression of two single guide RNAs (sgRNA) targeting the *BmPMFBP1* gene. The targets are required to be located on the exon of the *BmPMFBP1* gene and conform to the 5’-G(N_19_)NGG-3’ rule. CRISPR/Cas9 targets sequences were obtained by submitting full-length cDNA sequence to the CRISPRdirect website (https://crispr.dbcls.jp/). The sgRNA sequences and primers are listed in S1 Table. A mix of the transgenic plasmid, helper plasmids and piggyBac transposon mRNA were injected into 480 G0 eggs. The G0 moth were sib-mated or mated with wildtype (WT) moths to produce a total of 37 broods (G1). The G1progeny were scored for the presence of the red fluorescent marker using fluorescence microscopy (Nikon AZ100) and we obtained four broods containing *DsRed*-positive. The U6-sgRNA positive lines and nos-Cas9 line were crossed to obtain *BmPMFBP1* mutants in the heterozygous F1 progeny that were used in subsequent experiments.

### Mutagenesis analysis

Genomic DNA was extracted from the mutants at larva stages using standard SDS lysis-phenol treatment, incubated with Proteinase K in 56°C for 5 hours, then treated with RNase and purified. Gene-specific primer were designed upstream or downstream of two targets. Using 100ng genomic DNA as template to do PCR. The PCR product was purified and cloned to the PJET1.2 vector and sequenced.

### RNA isolation and quantitative real-PCR

Total RNA was extracted from the different tissues of three individual silkworms at L5D4 larval stage using TRIzol reagent. Subsequently the RNA was reverse-transcribed into cDNA using RT reagent Kit with gDNA Eraser. The mRNA expression levels of gene were detected using SYBR Green Real-time PCR Master Mix via being normalized relative to ribosomal protein *BmRP49*. The mRNA measurements were quantified in three independent biological replicates, each using three independent technical replicates.

### Observation of sperm bundles

Sperm bundles or sperm from different stage (fifth larval stage and pupa stage) were collected in 1.5ml tubes and were fixed in PBS with 4% paraformaldehyde for 1hour. The samples were washed three times using PBS, then the actin proteins were stained with TRITC Phalloidin for 1 hour, the nuclei were stained with Hoechst for 10 min. The samples were washed three times using PBS again, subsequently smeared on a microslide, and observed using fluorescence microscopy (Olympus, FV1000, Leica SR5).

### Paraffin section and hematoxylin eosin staining

The testes from mutants and wildtype were dissected and immediately preserved in fixative (anhydrous ethanol/acetic acid/chloroform, 6/1/3(vol/vol/vol)) for 24h, stored in 70% (vol/vol) ethanol, and dehydrated three times by anhydrous ethanol, then cleared three times using xylene. The samples were be embedded in paraffin overnight. The 5 μm sections were cut by Leica microtome (RM2235). After deparaffinization, the sections were stained with a mixture of hematoxylin and eosin for histological analysis. Pictures were analyzed and photographed with a microscope (Olympus BX53).

### Double copulation

In control single-copulation matings, either a *BmPMFBP1* or a *BmSxl* mutant male was mated with a wildtype virgin female for 3 hours. In the double-copulation group, a virgin female was first mated with the *BmPMFBP1* mutant male, followed the *BmSxl* mutant male. In this double mating, both males were previously mated a virgin female to ensure sterility.

### Statistical analysis

All data were analyzed by GraphPad Prism (version 5.01) and presented as ± SEM. The statistically significant differences were measured by Student’s t-test with a paired, 2-tailed distribution (*, P < 0.05; **, P < 0.01; ***, P < 0.001).

### Bioinformatic analyses

Starting with the X1 isoform for BmPMBFBP1 (NCBI RefSeq Gene 101744903), a BLASTn search was performed against the gene models in the SilkDB3 database [31]. Four genes with significant similarity were identified, all located in the region of Chromosome 4 corresponding to BmPMFBP1. Transcript abundance levels (FPKM) were downloaded from SilkDB3 and visualized using ggplot2 [44]. Two of the four identified genes had expression levels less than 3 FPKM for any tissue and were excluded from further evaluation.

We used the HMMer software [34] to perform a targeted sequence homology search in order to more sensitively detecting homologs of mammalian PMFBP1 in insect proteomes. To build a custom HMM profile, we used orthologous proteins (one per species) listed for PMFBP1 in the NCBI “Gene” database (https://www.ncbi.nlm.nih.gov/gene/83449/ortholog/). We specifically selected mammalian species (163 species), and excluded sequences <700 and >1500 bp on the assumption that these were misannotated, leaving 153 distinct species represented in the set of orthologs. These protein sequences were aligned with COBALT via the NCBI website (https://www.ncbi.nlm.nih.gov/tools/cobalt/re_cobalt.cgi; S2 Fig), and the resulting multiple sequence alignment was provided to HMMer via the EBI website (https://www.ebi.ac.uk/Tools/hmmer/) to generate an HMM profile and directly search the ensemblMetazoa database for insect taxa. We subsequently conducted taxon specific HMMsearches using the command line version of HMMer, assaying the complete set of predicted proteins for *B*. *mori* (RefSeq v103) and *Drosophila melanogaster* (FlyBase r6.40).

## Supporting information

S1 Fig Transcript annotations of *BmPMFBP1* locus. Graphical representation of relevant transcript annotations along the region of *B*. *mori* chromosome 4 corresponding to the PMFBP1 locus.
(TIF)

S2 Fig COBALT multiple sequence alignment of PMFBP1. COBALT multiple sequence alignment of PMFBP1 from 153 mammalian species, used as input for generating an HMM profile for homology searches in insects.
(TIF)

S3 Fig The deletion of *BmPMFBP1* did not affect the morphology of testes. (A) The images of testes of wildtype (WT) and *ΔBmPMFBP1* in L5D4. (B) The morphologies of internal structure of testes from WT and *ΔBmPMFBP1* in L5D4. The paraffin sections stained with hematoxylin and eosin.
(TIF)

S4 Fig The deletion of *BmPMFBP1* did not affect the apyrene sperm. (A and B) Fluorescence image of apyrene sperm bundles in testes of wildtype (WT) males and ΔBmPMFBP1 males on seventh day of pupa stage. The apyrene sperm bundles displayed normal morphology in ΔBmPMFBP1 males. (B) The stain of the nuclei in the apyrene sperm bundles. (C) The double copulation rescued the sterility caused by *BmPMFBP1* mutant. Fertility was evaluated as the ratio of fertile individuals to the total number of individuals (n = 15). The filamentous actin proteins were stained with TRITC Phalloidin, the nuclei were stained with Hoechst 33258.
(TIF)

S5 Fig The morphology of the sperm bundles in adults. (A-C) Fluoresence image of eupyrene sperm bundles (A), nucleate apyrene sperm bundles (B) and anucleate apyrene sperm bundles (C) in testes of wildtype (WT) males and *ΔBmPMFBP1* males on adult stage. The filamentous actin proteins were stained with TRITC Phalloidin, the nuclei were stained with Hoechst 33258.
(TIF)

S1 Table Primers used in this work. (DOCX)

S1 Data Raw data for Fig 1B. (XLSX)

S2 Data Raw data for Fig 2C. (XLSX)

S3 Data Raw data for Fig 3B. (XLSX)

S4 Data Raw data for Fig 3C. (XLSX)

S5 Data Raw data for S4C Fig. (XLSX)
