# Supplementary figures and images for: BmPMFBP1 regulates the development of eupyrene sperm in the silkworm, Bombyx mori
Source: PLoS Genet. 2022 Mar 21;18(3):e1010131. doi: 10.1371/journal.pgen.1010131 (PMC8970482; doi:10.1371/journal.pgen.1010131)

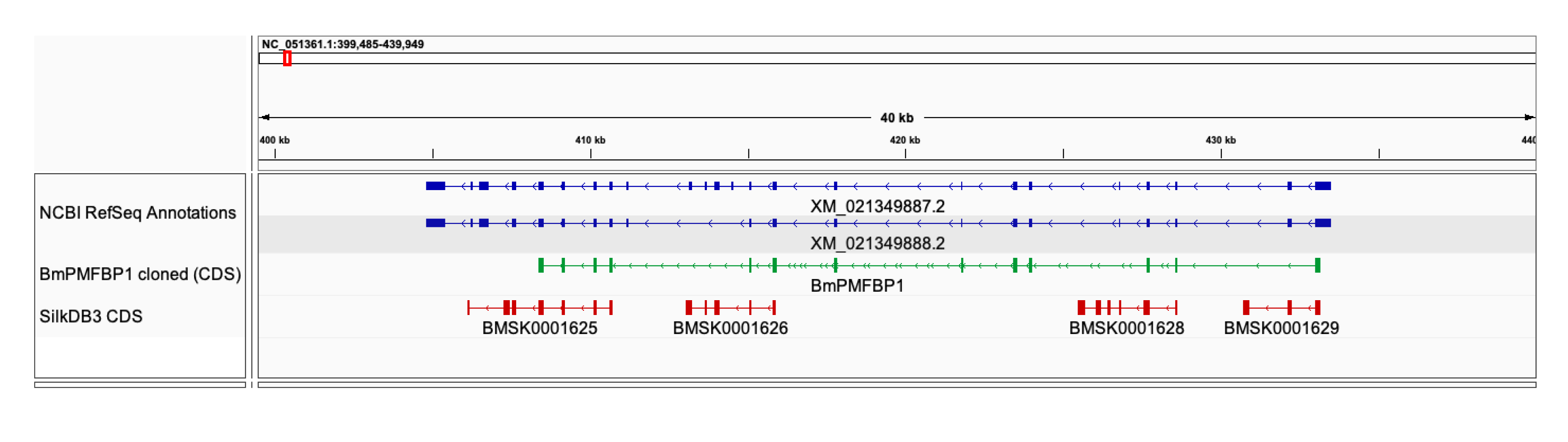

Supplement: S1 Fig — Graphical representation of relevant transcript annotations along the region of B. mori chromosome 4 corresponding to the PMFBP1 locus. (TIF) [file pgen.1010131.s001.tif]

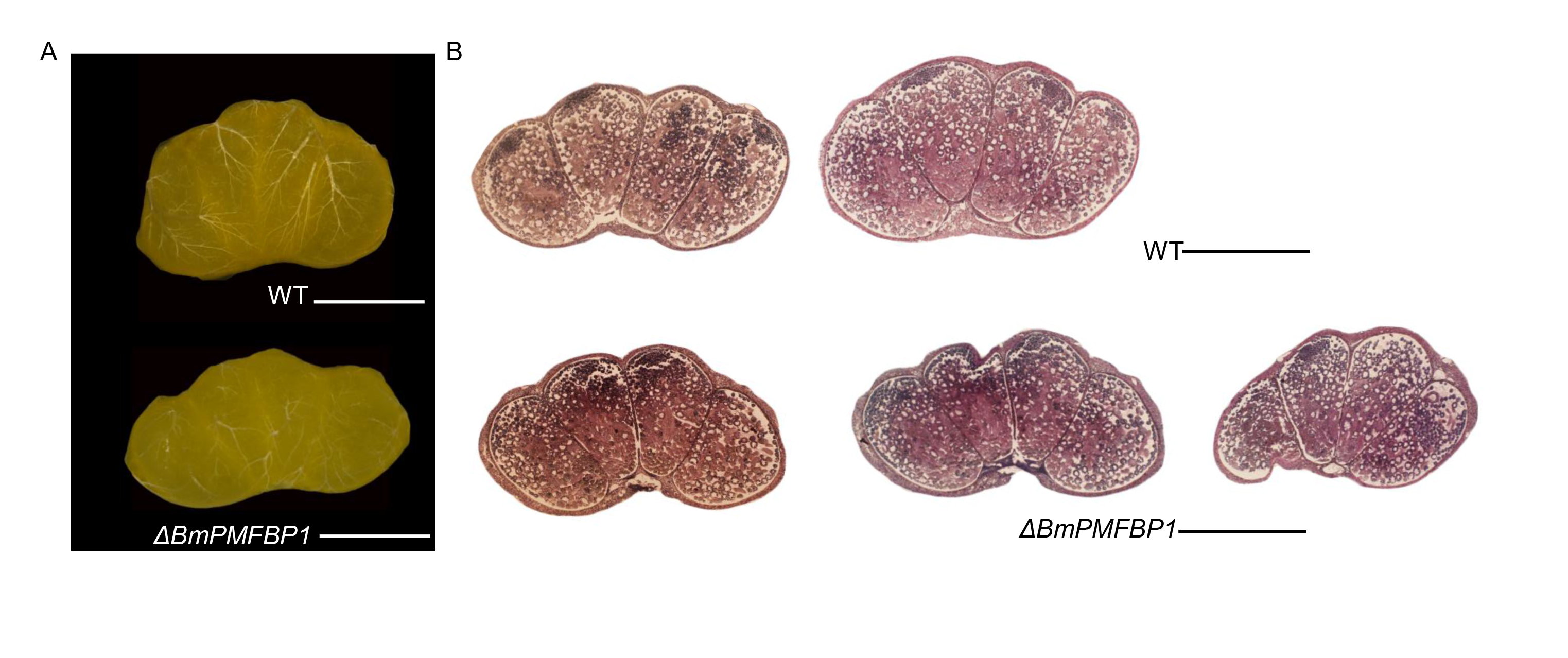

Supplement: S3 Fig — (A) The images of testes of wildtype (WT) and ΔBmPMFBP1 in L5D4. (B) The morphologies of internal structure of testes from WT and ΔBmPMFBP1 in L5D4. The paraffin sections stained with hematoxylin and eosin. (TIF) [file pgen.1010131.s003.tif]

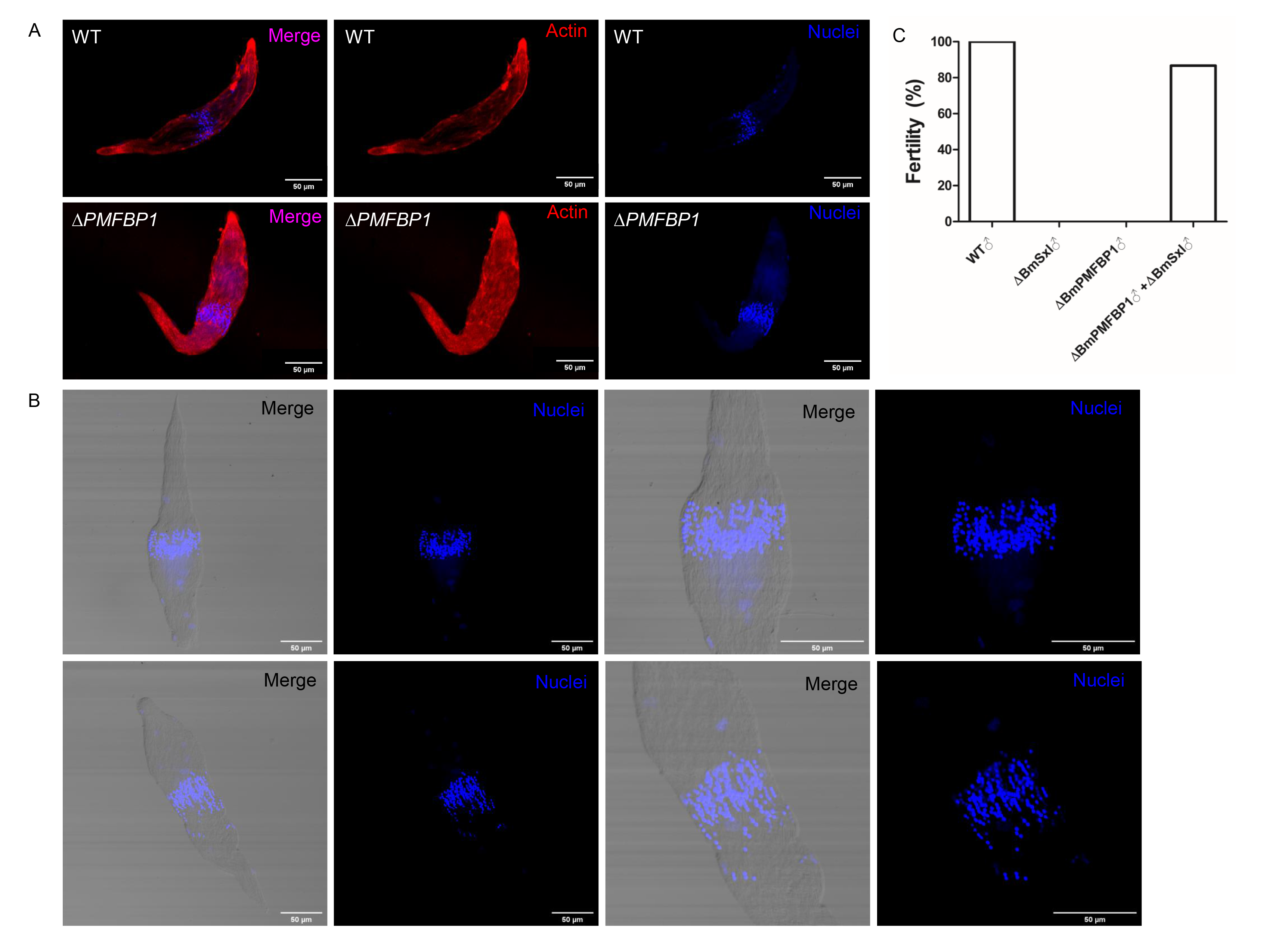

Supplement: S4 Fig — (A and B) Fluorescence image of apyrene sperm bundles in testes of wildtype (WT) males and ΔBmPMFBP1 males on seventh day of pupa stage. The apyrene sperm bundles displayed normal morphology in ΔBmPMFBP1 males. (B) The stain of the nuclei in the apyrene sperm bundles. (C) The double copulation rescued the sterility caused by BmPMFBP1 mutant. Fertility was evaluated as the ratio of fertile individuals to the total number of individuals (n = 15). The filamentous actin proteins were stained with TRITC Phalloidin, the nuclei were stained with Hoechst 33258. (TIF) [file pgen.1010131.s004.tif]

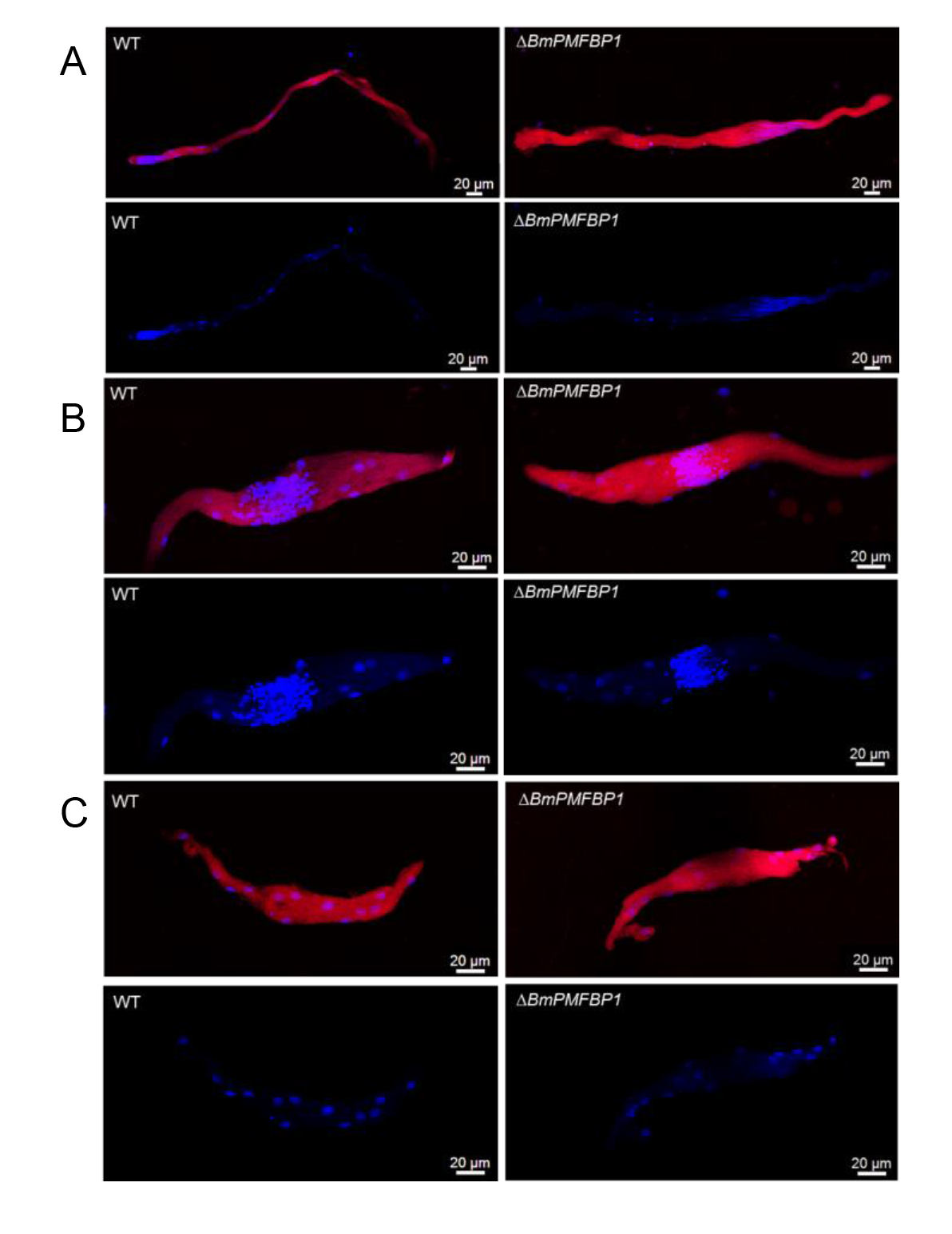

Supplement: S5 Fig — (A-C) Fluoresence image of eupyrene sperm bundles (A), nucleate apyrene sperm bundles (B) and anucleate apyrene sperm bundles (C) in testes of wildtype (WT) males and ΔBmPMFBP1 males on adult stage. The filamentous actin proteins were stained with TRITC Phalloidin, the nuclei were stained with Hoechst 33258. (TIF) [file pgen.1010131.s005.tif]
