# Supplementary material for: BmPMFBP1 regulates the development of eupyrene sperm in the silkworm, Bombyx mori
Source: PLoS Genet. 2022 Mar 21;18(3):e1010131. doi: 10.1371/journal.pgen.1010131 (PMC8970482; doi:10.1371/journal.pgen.1010131)
Supplement: S1 Table — (DOCX) [file pgen.1010131.s006.docx]

**S1 Table**

| Primer name | Primer sequence(5’to3’) | The purpose |
| --- | --- | --- |
| BmPMFBP1 F | ATGAATCAAAATATAATAGATTGTGATGCC | ORF |
| BmPMFBP1 R | TCAGGCCTCCCGCGCGCGCG | ORF |
| BmPMFBP1-sg1-F | AATATCGTGCTCTACAAGTgACCCCGGCCTCTTCGATCCGTTTTAGAGCTAGAAATAGC | KO Plasmid construction |
| BmPMFBP1-sg1-R | CCTGAAGTCGTGGGTGAGCACTTGTAGAGCACGATATT | KO Plasmid construction |
| BmPMFBP1-sg2-F | CTCACCCACGACTTCAGGAGTTTTAGAGCTAGAAATAG | KO Plasmid construction |
| Sg2-R | AACCTtatcgataccgtcgaAAAAAAAAGCACCGACTCGG | KO Plasmid construction |
| BmPMFBP1-KO-F | ATGGGAAACGGTTGCTTTTCGT | Mutagenesis analysis |
| BmPMFBP1-KO-R | GACTGACGACTGAGCGGTCTTA | Mutagenesis analysis |
| RP49 F | TCAATCGGATCGCTATGACA | q-RT-PCR |
| RP49 R | ATGACGGGTCTTCTTGTTGG | q-RT-PCR |
| BmPMFBP1-qPCR-F | GCCGGGGTCGGGAATAAGAC | q-RT-PCR |
| BmPMFBP1-qPCR-R | CCTCTACAGCCTGCACGAGC | q-RT-PCR |
| IE1 EGFP F | TGACTGGCGGCGACAAGAGGTACCGTGAGCAAGGGCGAGGAGCT | EGFP Plasmid construction |
| EGFP SV40 R | TATCTAGATCCGGTGGATCCGGTACCCTACTTGTACAGCTCGTCCA | EGFP Plasmid construction |
| IE1 PMFBP1 F | TGACACTGGCGGCGACAAGAGGTACCATGAATCAAAATATAATAGA | PMFBP1 EGFP Plasmid construction |
| IE1 PMFBP1R | AGCTCCTCGCCCTTGCTCACGGCCTCCCGCGCGCGCGCCC | PMFBP1 EGFP Plasmid construction |
| EGFP1 F | GTGAGCAAGGGCGAGGAGCT | PMFBP1 EGFP Plasmid construction |
